# The economical role of RIOK3 in modulating the Jak1/STAT1 pathway and antiviral immunity against respiratory syncytial virus infection in macrophages: implications for therapeutic potential

**DOI:** 10.3389/fmicb.2025.1591473

**Published:** 2025-04-30

**Authors:** Fangfang Sun, Weiwei Liang, Yingying Qian, Pengfei Hu

**Affiliations:** ^1^Department of Colorectal Surgery and Oncology, Key Laboratory of Cancer Prevention and Intervention, Ministry of Education, The Second Affiliated Hospital, Zhejiang University School of Medicine, Hangzhou, China; ^2^Cancer Center, Zhejiang University, Hangzhou, China; ^3^Department of Endocrinology, The Second Affiliated Hospital, Zhejiang University School of Medicine, Hangzhou, China; ^4^Department of Geriatrics, Second Affiliated Hospital, School of Medicine, Zhejiang University, Hangzhou, China; ^5^Department of Orthopedic Surgery, Second Affiliated Hospital, School of Medicine, Zhejiang University, Hangzhou, China; ^6^Orthopedics Research Institute of Zhejiang University, Hangzhou, China

**Keywords:** RSV, macrophage, RIOK3, Jak1/STAT pathway, type I interferon

## Abstract

Respiratory Syncytial Virus (RSV) is a leading cause of lower respiratory tract infections, particularly in vulnerable populations such as infants, the elderly, and immunocompromised individuals. RSV infection can result in mortality rates as high as 20%, attributable not only to viral replication but also to an excessive host immune response. Current therapeutic options are limited, partly due to gaps in understanding the host immune response, especially the role of macrophages and their signaling pathways. This study investigates the role of RIOK3, an unconventional kinase, in modulating the Jak1/STAT1 pathway during RSV infection in macrophages and its impact on viral replication and interferon production. Using both *in vitro* and *in vivo* models, including primary bone marrow-derived macrophages (BMM) from control and RIOK3 knockout (KO) mice, we demonstrate that RIOK3 is a critical regulator of the Jak1/STAT1 pathway in macrophages during RSV infection. The absence of RIOK3 enhances viral replication and disrupts the balance of type I interferons. Targeting RIOK3 may represent a promising strategy to enhance antiviral immunity and mitigate RSV-induced inflammation, thus warranting further investigation for therapeutic potential.

## 1 Introduction

Respiratory Syncytial Virus (RSV) is an enveloped, negative-sense, single-stranded RNA virus that poses a significant threat to public health, particularly among infants, the elderly, and immunocompromised individuals (Zhao et al., 2024). Since its discovery in the 1950s, RSV has been the most common viral pathogen of lower respiratory tract infections in infants and young children worldwide, accounting for 50% to 80% of hospitalizations for bronchiolitis and 25% of pneumonia in infants and young children each year (Guo et al., 2024; Meissner, 2016). Moreover, RSV infection is associated with a 20% mortality rate in affected patients, and survivors are at an increased risk of developing bronchial asthma later in life (Sigurs et al., 2010). This outcome has been attributed to massive immune cell infiltration and the production of inflammatory cytokines, which can cause irreversible lung damage (Balhara and Gounni, 2012; Jartti and Gern, 2017).

During the natural course of RSV infection, alveolar macrophages, which are part of the innate immune system, are the first line of defense against the virus. They are thought to be critical throughout the course of virus infection, including activation, antigen presentation to immune cells, cytokine release, pathogen killing, and tissue repair. Concerning the long-term effects of macrophage activation, several studies have found that macrophages alter the intensity of the inflammatory response, which has been linked to immune-sensitization and the pathology of airway hyperresponsiveness in RSV-infected infants in their late life (Wang et al., 2022). Type I interferons (IFNs) are the major cytokines released by activated macrophages and are essential for host defense against viral infections. They limit viral replication in infected cells and promote antiviral immune responses (Crouse et al., 2015). However, the Type I IFNs system has also been implicated in severe lung inflammation in response to RSV infection (Ansar et al., 2021). This raises a critical question: How do host cells efficiently direct the immune response against the virus while minimizing potential pathological inflammation via macrophages?

We previously identified RIOK3, a phosphorylating kinase, as a pivotal regulator of viral infection in macrophages through siRNA screening (Feng et al., 2014). As an unconventional kinase, RIOK3 is expressed exclusively in eukaryotes (Anaya et al., 1998) and is highly expressed in myeloid cells (Uhlen et al., 2015). Several studies have highlighted the multifaceted role of RIOK3 in immune-related cellular signaling. For instance, RIOK3 has been shown to alternatively regulate the NF-κB signaling pathway (Liu et al., 2025; Bisom et al., 2023; Shan et al., 2009), attenuate the innate immune response by mediating the phosphorylation of MDA5 (Takashima et al., 2015), and modulate IRF3-mediated antiviral Type I IFNs production (Feng et al., 2014). Our group has further demonstrated that RIOK3 regulates Type I IFNs production by modulating the ubiquitination and subsequent degradation of RIG-I and MDA5, thereby negatively regulating the RLR-mediated signaling pathway (Shen et al., 2021). This interaction helps maintain the balance of IFN production in the innate immune response and prevents overactivation. However, despite these insights, the specific role of RIOK3 in RSV infection and its interaction with the JAK1/STAT1 pathway, a key downstream effector of the IFN response, remains unclear.

Given the established roles of RIOK3 in immune signaling and the importance of macrophages in RSV infection, we hypothesize that RIOK3 may significantly modulate the immune response to RSV infection in macrophages. Specifically, we aim to investigate whether RIOK3 participates in the JAK1/STAT1 pathway, which is central to the interferon response to viral infections. By elucidating these mechanisms, we hope to further understand how the host regulates the immune and inflammatory balance during viral infection and contribute to the development of novel therapeutic strategies against RSV.

## 2 Materials and methods

### 2.1 Cell culture and reagents

Primary bone marrow-derived macrophages (BMM) were isolated from control and RIOK3 knockout mice (LysMCre+Riok3^F/F^), and cultured in RPMI 1640 medium supplemented with 10 ng/ml M-CSF for 7 days. Detailed information regarding mouse generation and culture conditions is referred to in our previous study (Shen et al., 2021). Previous published research has demonstrated that the deletion efficiency of LysMCre mice model in mature macrophages is about 83%−98% (Clausen et al., 1999). The HEp-2, HEK293T, THP-1, and Vero cell lines were originally obtained from ATCC and cultured in RPMI 1640 medium supplemented with 10% fetal bovine serum (FBS), 100 U/ml penicillin, and 100 μg/ml streptomycin at 37°C in a 5% CO_2_ incubator.

### 2.2 Viral infection and titration

Respiratory Syncytial Virus (RSV) stocks (RSV-A strain Long) were propagated in HEp-2 cells as previously described (Wang et al., 2015). Virus titers were determined by plaque assay on HEK293T cells (Wang et al., 2015), and aliquots were stored at −80°C until use. For macrophage infections, THP-1 cells and BMMs from control and RIOK3-KO mice were infected with RSV at a multiplicity of infection (MOI) of 5. Infections were performed in RPMI 1640 without FBS, and cells were incubated at 37°C for 2 h with periodic rocking to ensure virus attachment. After adsorption, the inoculum was replaced with RPMI 1640 containing 2% FBS, and cells were further incubated for the indicated hours post-infection (hpi). The *in vivo* infection protocol is detailed in our previous study (Shen et al., 2021). Briefly, age- and sex-matched littermate mice were selected to ensure experimental consistency. Intranasal infection was performed with an RSV titer of 1 × 10^7^ pfu/mouse to assess infection levels. Post-infection, mice were closely monitored for signs of infection and adverse effects. Lung tissue and lavage samples were collected at set intervals for viral infection assessment.

### 2.3 Quantitative real-time PCR

Total RNA was extracted from infected cells using the Ultrapure RNA Kit (CWbiotech). Reverse transcription was performed using the HiFiScript 1st Strand cDNA Synthesis Kit (CWbiotech) to synthesize cDNA. qPCR was conducted using iTaq Universal SYBR Green Supermix (Bio-Rad) to quantify *RSV-F* gene expression and host immune response genes (Ifn-α, Ifn-β, Il6, Il1-β, Tnf-α, and ISGs) on the CFX96 Touch Real-Time PCR Detection System (Bio-Rad). Relative mRNA expression was calculated using the 2^−^ΔΔCt method, normalized to β-actin. Primer sequences are listed in Supplementary Table 1.

### 2.4 ELISA

Lavage and supernatants from infected macrophages were collected at various time points and analyzed for IFN-α, IFN-β, IL-6, IL-1β, and TNF-α using enzyme-linked immunosorbent assay (ELISA) kits (eBioscience) according to the manufacturer's instructions.

### 2.5 Western blotting and immunoprecipitation

Protein lysates were prepared from infected macrophages using RIPA buffer. Protein concentrations were determined using the BCA Protein Assay (CWbiotech). Equal amounts of protein were separated by SDS-PAGE and transferred to PVDF membranes. Membranes were blocked and incubated with primary antibodies against RIOK3, phospho-JAK1, JAK1, phospho-STAT1, STAT1, and β-actin (detailed information on antibodies is listed in Supplementary Table 1). After incubation with HRP-conjugated secondary antibodies, bands were visualized using a chemiluminescent substrate. For immunoprecipitation (IP), cell lysates were incubated with anti-JAK1 antibody overnight at 4°C with protein A/G magnetic beads. Beads were washed three times with lysis buffer, and immunoprecipitated JAK1 was eluted. For immunoblotting, JAK1 and RIOK3 were detected using respective antibodies, followed by visualization with appropriate secondary antibodies and an imaging system.

### 2.6 Plasmid construction and transfection

**Figure 1:**
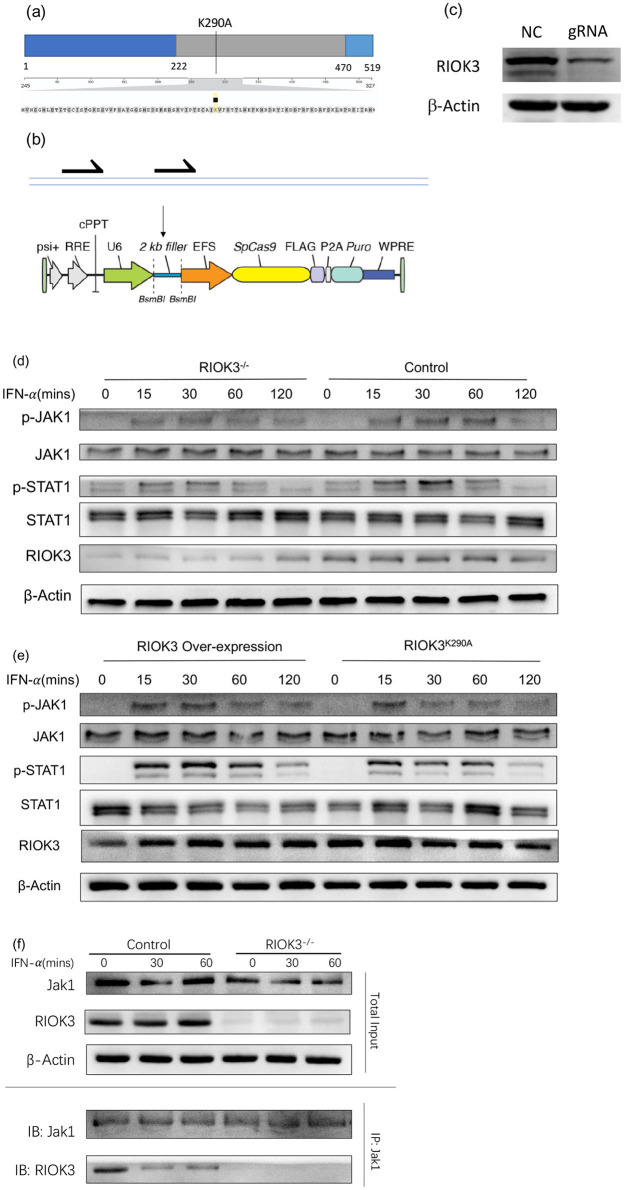
RIOK3 enhances JAK1/STAT1 pathway activity by interacting with Jak1 partially depend of its kinase function. **(a, b)** Sketch maps for kinase-dead RIOK3^K290A^ plasmid and gRNA inserted CRISPR/Cas9 plasmid construction. **(c)** Vero cells were transfected with gRNA-inserted plasmid or negative control for 48 h. Cell protein was assayed by WB to detect RIOK3 expression. **(d)** Vero cells with or without RIOK3-KO were stimulated with human IFN-α (1,000 units/ml) for 0, 15, 30, 60, and 120 min. Protein was collected for WB to analyze expression level of phosphor-Jak1, total Jak1, phospho-STAT1, total STAT1, RIOK3, and β-actin. **(e)** RIOK3-KO Vero cells were transfected with RIOK3 Over-expression plasmid or Kinase Dead RIOK3^K290A^ plasmid for 48 h, and then were stimulated with human IFN-α (1,000 units/ml) for 0, 15, 30, 60, and 120 min. Protein was collected for WB to analyze expression level of phosphor-Jak1, total Jak1, phospho-STAT1, total STAT1, RIOK3, and β-actin. **(f)** BMMs from control or RIOK3^−/−^ mice were stimulated with murine IFN-α (100 units/ml) for 0, 15, and 30 min. Protein was collected and divided into two parts. One was assayed directly to display the total input volume of RIOK3, Jak1, and β-Actin protein by WB. One was performed immunoprecipitation using Jak1 antibody and both Jak1 and RIOK3 were assayed by WB.

To generate RIOK3-KO Vero cells, CRISPR/Cas9 plasmids containing a guide RNA (gRNA) targeting RIOK3 or a negative control (NC) gRNA were constructed according to the schematic diagram (Figure 1b). Vero cells were transfected with these plasmids using Lipofectamine 2000 according to the manufacturer's instructions (Invitrogen). After 48 h post-transfection, successful RIOK3 knockout was confirmed by Western blotting (Figure 1c). Remaining transfected cells were selected with puromycin to obtain pooled RIOK3-KO Vero cells. The original CRISPR/Cas9 plasmid vector was obtained from Addgene. The overexpression and kinase-dead mutant RIOK3K290A plasmids were obtained from Dr. Feng's Lab (Feng et al., 2014) (Figure 1a).

### 2.7 Histopathology

On days 3 and 5 post-infection, mice were euthanized to harvest lung tissues and lavage. Tissues were fixed in 10% buffered formalin, followed by paraffin embedding for more than 48 h. Cross-sections were stained with hematoxylin and eosin (H&E). Light microscopy was used to capture images at 4X and 40X magnifications.

### 2.8 Ethical approval statement

All animal experiments were conducted in accordance with the ethical standards and approval granted (approved research proposal number 2020-414) by the Animal Ethics Committee of the Second Affiliated Hospital of Zhejiang University School of Medicine. An overdose injection of barbiturate was used as the method of euthanasia.

### 2.9 Statistical analysis

Data are presented as mean ± standard error of the mean (SEM) from at least three independent experiments. Statistical significance was determined using a two-tailed Student's *t*-test or one-way ANOVA with Tukey's *post-hoc* test, as appropriate. A *p*-value of < 0.05 was considered statistically significant. Statistical analysis was performed using GraphPad Prism 9 (GraphPad Software, Inc., San Diego, CA, USA).

## 3 Results

### 3.1 Expression of RIOK3 is dynamically increasing in macrophages during RSV infection

**Figure 2:**
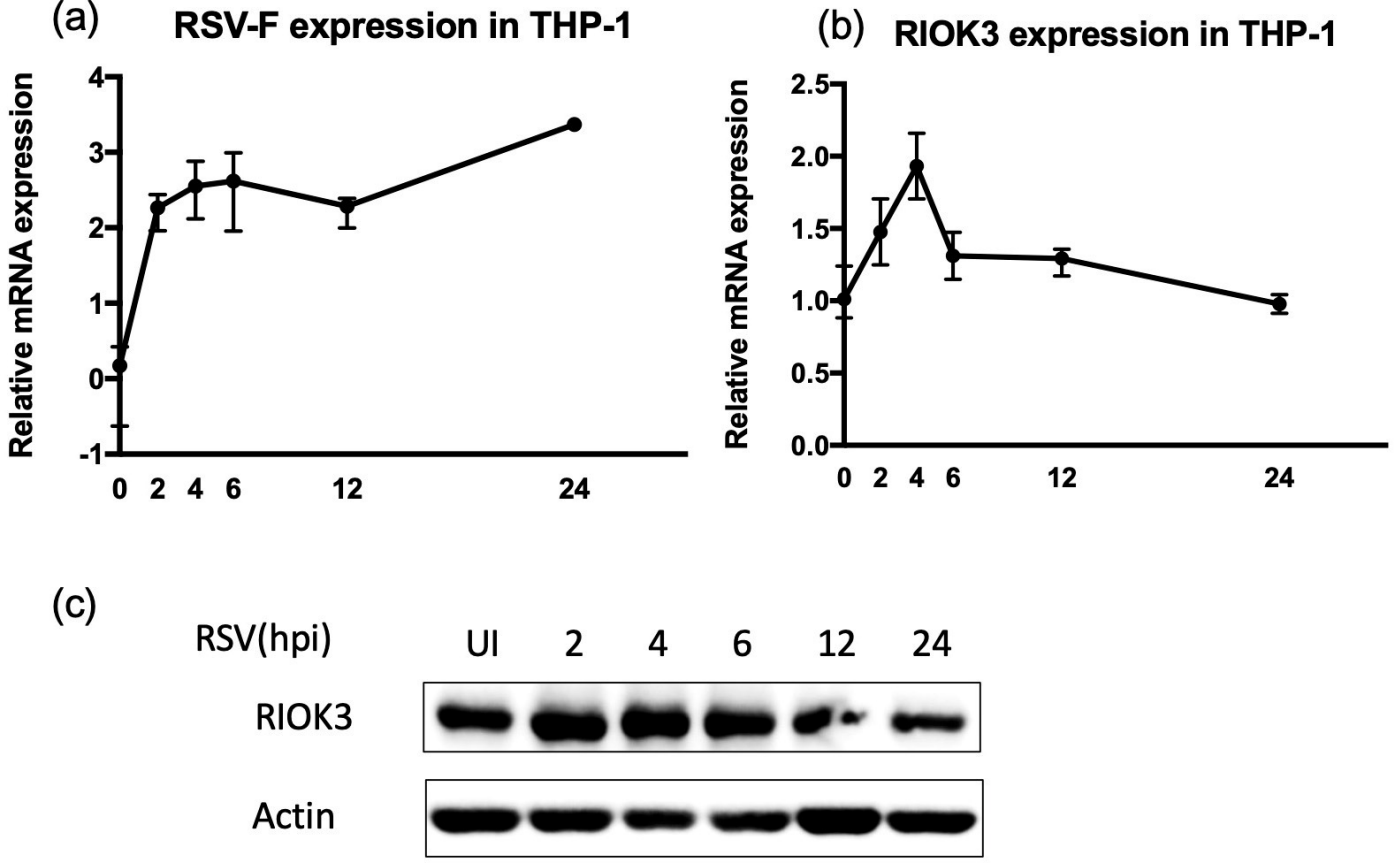
Expression of RIOK3 is dynamically increasing in macrophages during RSV infection. THP1 cells were dynamically infected with RSV for 0, 2, 4, 6, 12, and 24 h at MOI = 5. Cells were harvested to assess mRNA expression of RSV-F **(a)** and RIOK3 **(b)** by qRT-PCR analysis, as well as dynamical change of RIOK3 protein level by WB **(c)**.

Our initial analysis focused on the expression levels of RIOK3 in macrophages following RSV infection using THP-1 cells. As RSV-F expression increased over time, a significant upregulation of RIOK3 mRNA was observed at 2-, 4-, and 6-h post-infection (hpi) compared to uninfected controls (Figures 2a, b). Meanwhile, Western blot (WB) results revealed a slight increase in RIOK3 protein levels at early time points, followed by a decrease at later stages of infection (Figure 2c).

### 3.2 Lack of RIOK3 is associated with enhanced expression both in RSV-f and type I IFN during infection

**Figure 3:**
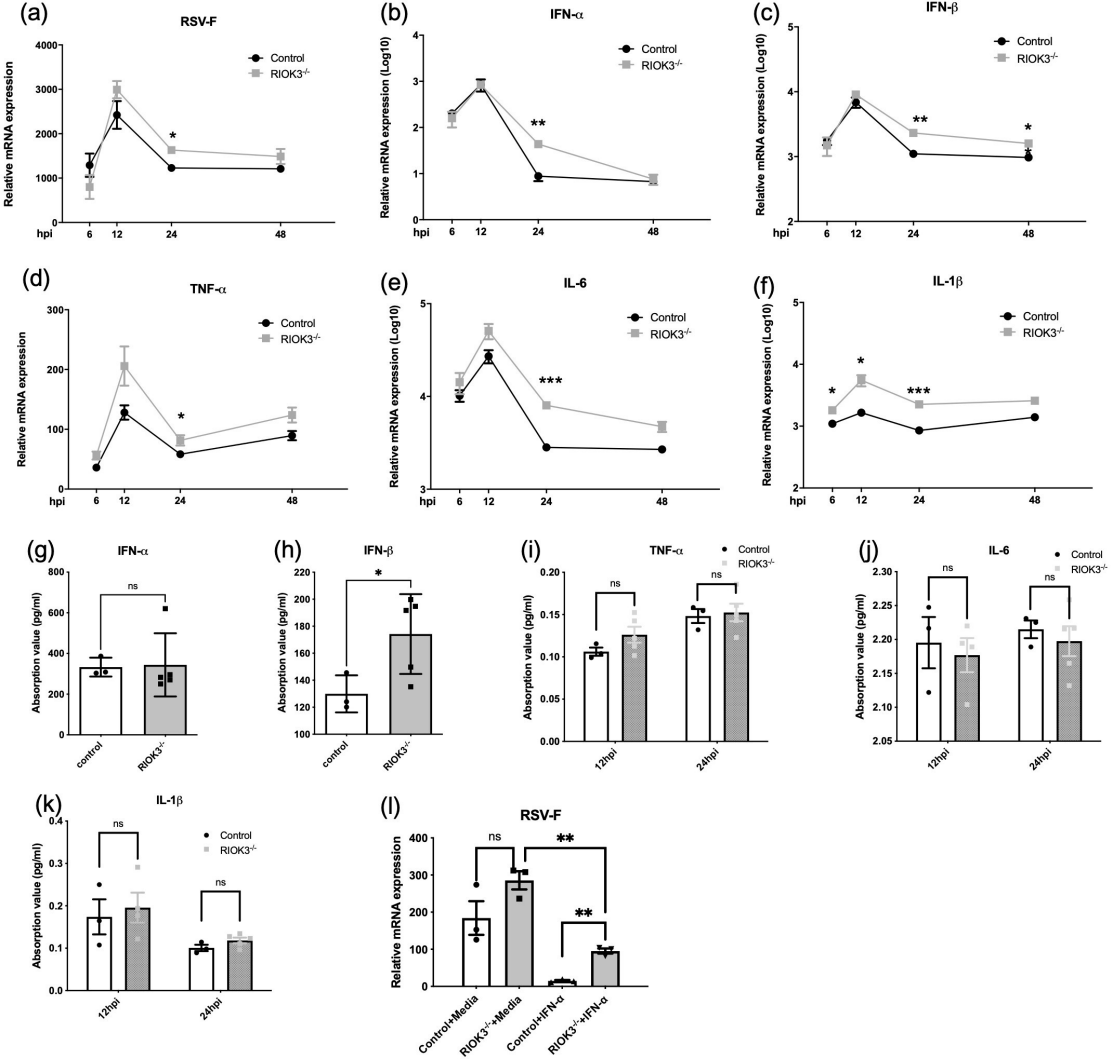
Lack of RIOK3 is associated with enhanced expression both in RSV-F and type I IFNs during infection. BMM from control or RIOK3^−/−^ mice were infected with RSV at MOI = 5. At 6, 12, 24, and 48 hpi, RNA was collected for qRT-PCR **(a–f)**, and supernatants were collected for plaque assay (failed) and ELISA **(g–k)**. **(g, h)** only showed ELISA results of indicated factor at 24 hpi. **(l)** BMMs from control or RIOK3^−/−^ mice were stimulated with or without murine IFN-α (100 units/ml) for 6 h and then infected with RSV at MOI = 5. At 12 hpi, cells were collected for RNA extraction and RSV-F mRNA level was assessed by qRT-PCR. Data are expressed as means ± SEM. Significance was determined using Student's *t*-test; *** *p* < 0.001, ** *p* < 0.01, and * *p* < 0.05 relative to non-infected cells.

To assess the impact of RIOK3 on RSV replication, we measured RSV-F mRNA expression, type I interferons (IFN-α and IFN-β) production, and several inflammatory factors in control and RIOK3-KO macrophages at various time points during viral infection. RSV-F mRNA expression was significantly higher in RIOK3-KO macrophages at 24 hpi, indicating enhanced viral replication in the absence of RIOK3 (Figure 3a). However, consistent with previous studies demonstrating restricted RSV replication in macrophages (Cirino et al., 1993; Rivera-Toledo and Gomez, 2012), we were unable to detect active viral particles by plaque assays.

Surprisingly, qPCR results showed that IFN-α and IFN-β mRNA levels, as well as inflammatory factors (IL-6, IL-1β, and TNF-α), were significantly elevated in RIOK3-KO macrophages compared to controls at 24 hpi (Figures 3b–f). ELISA analysis revealed a consistent increase in IFN-β protein production in RIOK3-KO macrophages at 24 hpi (Figures 3g–k). These findings suggest that RIOK3 may play a role in modulating type I IFNs induction following viral infection, as previously demonstrated by our group (Shen et al., 2021).

This presents a paradox: the absence of RIOK3 not only enhances RSV replication but also increases the production of antiviral factors (type I IFNs). To clarify the role of RIOK3 in the downstream signaling pathway of type I IFNs, bone marrow-derived macrophages (BMMs) were primed with IFN-α for 6 h before RSV infection. The results showed that RIOK3 knockout significantly enhanced RSV replication in macrophages primed with IFN-α (Figure 3l).

While we did observe an increase in RSV replication and type I IFNs production in our *in vitro* virus infection experiments, these phenotypes were not consistently observed in our *in vivo* experiments, as shown in Supplementary Figures 1a–e. This discrepancy may be attributed to the specific transgenic mouse model we used (LyzMCre mice). In this model, RIOK3 is selectively knocked out in myeloid cells, with a deletion efficiency of approximately 83%−98% in macrophages. This partial knockout may have influenced the overall immune response and viral replication dynamics *in vivo*. It also implied that the influence of RIOK3 expressed in macrophages is not strong enough to leverage the whole virus infection course.

### 3.3 Lack of RIOK3 inhibit activation of jak1/STAT1 pathway

**Figure 4:**
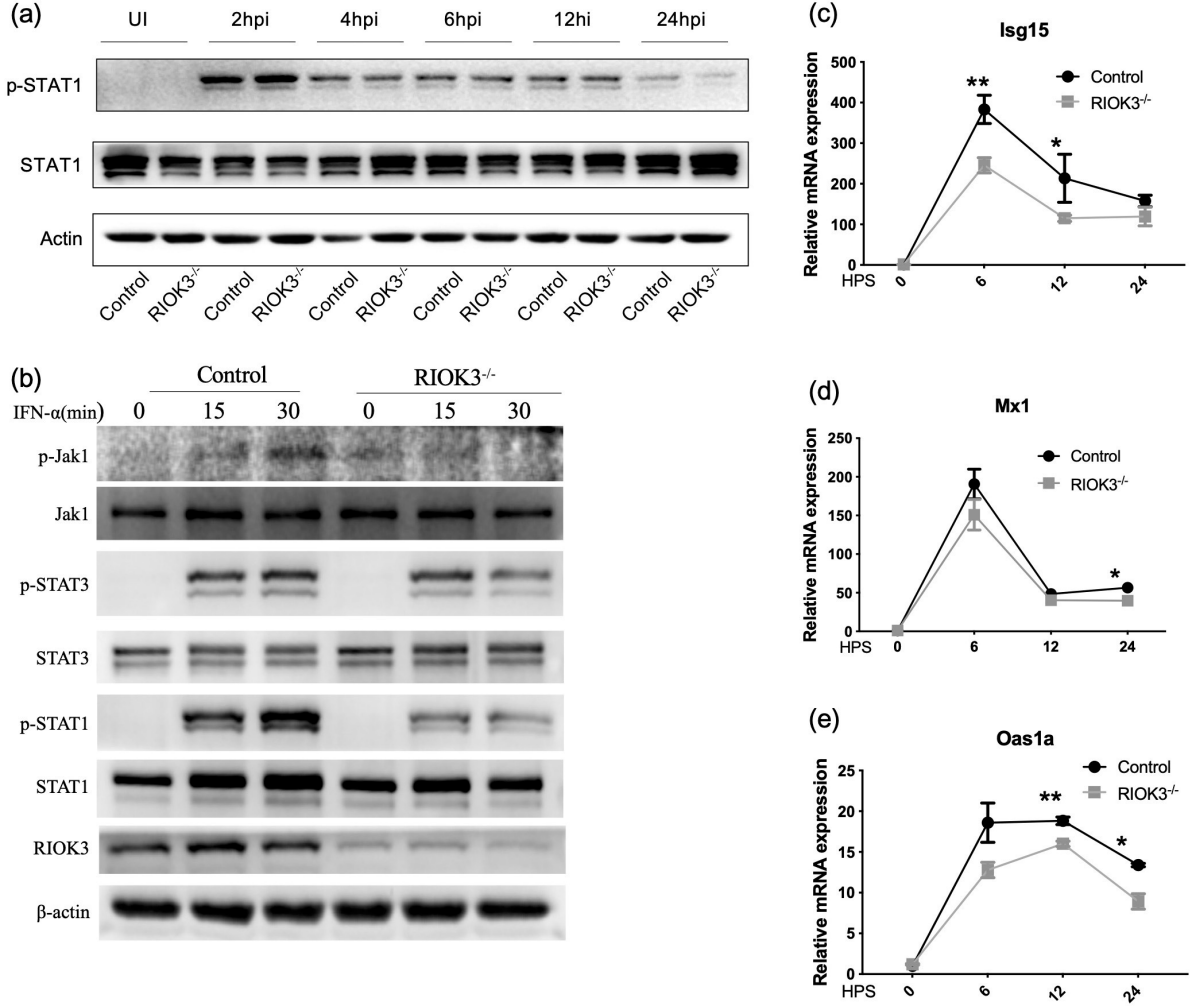
Lack of RIOK3 inhibit activation of Jak1/STAT1 pathway. **(a)** BMMs from control or RIOK3^−/−^ mice were infected with RSV at MOI = 5. At 0 (un-infection, UI), 2, 4, 6, 12, and 24 hpi, protein was collected for WB. Phospho-STAT1, total STAT1 and β-actin was assayed. **(b)** BMMs from control or RIOK3^−/−^ mice were stimulated with murine IFN-α (100 units/ml) for 0, 15, and 30 min. Protein was collected for WB to analyze expression level of phosphor-Jak1, total Jak1, phospho-STAT1, total STAT1, phospho-STAT3, total STAT3, RIOK3, and β-actin. **(c–e)** BMMs from control or RIOK3^−/−^ mice were stimulated with murine IFN-α (100 units/ml) for 0, 6, 12, and 24 h. RNA was collected to assess ISGs expression, including Isg15, Oas1, and Mx1. Data are expressed as means ± SEM. Significance was determined using Student's *t*-test; ** *p* < 0.01 and * *p* < 0.05 relative to non-stimulated cells.

To elucidate the impact of RIOK3 on the downstream signaling pathway of type I IFNs, primarily the JAK1/STAT1 pathway, we performed Western blot analysis during RSV infection. Results showed attenuated phosphorylation of STAT1 in RIOK3-KO macrophages at 24 hpi (Figure 4a). While the effect is not uniform across all time points, the consistent observation at 24 h supports the conclusion of diminished STAT1 activation in the KO cells. To eliminate the confounding effect of elevated IFNs production and focus solely on the downstream signaling pathway, BMMs were treated with IFN-α. Western blot results confirmed that phosphorylation of JAK1, STAT1, and STAT3 (not significant) was diminished in RIOK3-KO macrophages at 15 and 30 min post-IFN-α stimulation (Figure 4b).

To further investigate the antiviral response, we measured the expression of interferon-stimulated genes (ISGs) including Isg15, Oas1a, and Mx1 in infected macrophages. Expression of these ISGs was significantly reduced in RIOK3-KO macrophages compared to controls at 6 h (Isg15), 12 h (Isg15, Oas1a), and 24 h (Oas1a, Mx1) post-IFN-α stimulation (Figures 4c–e). These findings indicate that RIOK3 is crucial for inducing an effective antiviral gene expression program by enhancing the JAK1/STAT1 pathway.

### 3.4 RIOK3 enhances JAK1/STAT1 pathway activity by interacting with jak1 partially depend of its kinase function

Given the role of type I IFNs in activating the JAK1/STAT1 pathway and the deficiency of IFNs production in Vero cells (Matskevich et al., 2009), we assessed the phosphorylation of JAK1 and STAT1 in control, RIOK3-KO, RIOK3-overexpression, and kinase-dead RIOK3 (RIOK3^K290A^) Vero cells following IFN-α stimulation. Consistent with previous results, reduced phosphorylation of JAK1 and STAT1 was observed in RIOK3-KO Vero cells compared to controls, confirming that RIOK3 is involved in the downstream signaling pathway of IFN-α (Figure 1d). Overexpression of RIOK3 in RIOK3-KO Vero cells restored JAK1 and STAT1 phosphorylation, while the kinase-dead mutant RIOK3^K290A^ partially failed to do so, highlighting the importance of RIOK3 kinase activity in this process (Figure 1e).

To further elucidate the mechanism by which RIOK3 interacts with the JAK1/STAT1 pathway, we performed immunoprecipitation experiments. We found that RIOK3 physically associates with JAK1, and this interaction is enhanced following IFN-α stimulation (Figure 1f). These results suggest that RIOK3 may directly modulate JAK1 activity.

## 4 Discussion

Our findings demonstrate that RIOK3 plays a significant role in modulating the host response to RSV infection in macrophages. We observed an increase in RIOK3 mRNA expression following RSV infection, suggesting that RIOK3 may be part of the innate immune response to the virus. The enhanced RSV replication in RIOK3-KO macrophages, as evidenced by increased RSV-F mRNA expression, indicates that RIOK3 may function as a restriction factor in RSV replication.

Macrophages, as key players in the innate immune system, interact with RSV in complex ways that influence infection outcomes and disease pathology. Our results reveal that viral replication appears to be reproducibly by detecting RSV-F expression, but not dramatically higher in the RIOK3-KO macrophages. However, consistent with previous studies demonstrating restricted RSV replication in macrophages (Cirino et al., 1993; Rivera-Toledo and Gomez, 2012), active viral particles are unable to be detected by plaque assays. The efficiency of viral entry into macrophages and subsequent release of new version can be influenced by various factors. Firstly, the differentiation and maturation state of macrophages influence their susceptibility to RSV infection and subsequent viral replication. *In vitro* differentiation of macrophages prior to infection results in a significant decrease in the number of cells that replicate the virus (Nikitina et al., 2018). Moreover, from the view of viral factors, RSV encodes several proteins that can modulate host cell functions and immune responses to achieve persistent infection, such as the non-structural proteins NS1 and NS2 by inhibiting interferon production and signaling (Spann et al., 2004). However, this also means that the virus has to balance between evading immunity and replicating efficiently within the host cell.

Consistent with our previous study (Shen et al., 2021), the increased production of IFN-α and IFN-β in RIOK3-KO macrophages highlights the importance of RIOK3 in negatively regulating the induction of type I IFNs following viral infection. As previously established, type I IFNs are crucial for establishing an antiviral state and modulating immune responses (Casanova and Abel, 2021). The dual role of type I IFNs in chronic viral infections and cancer has been extensively discussed, with these cytokines exhibiting both proinflammatory and immunosuppressive effects (Lee-Kirsch, 2017; Snell et al., 2017). Another study revealed that RSV infection induces lung inflammation and oxidative stress via type I interferon signaling (Ansar et al., 2021). Our data present a paradox: despite increased IFNs production in the absence of RIOK3, RSV replication was slightly enhanced, suggesting that RIOK3 is necessary for an effective type I IFNs response. We have observed that the effect of diminished STAT1 phosphorylation in the RIOK3-KO cells is most pronounced at the 24-h post virus infection. This does not only because the interference of increased IFNs production during virus infection, but also relies on the joint action of virus factors. Former studies have revealed that RSV has evolved various strategies to evade host immune responses. For instance, RSV-infected macrophages produce type I IFNs but fail to respond to it in an autocrine manner due to impaired STAT1 phosphorylation by host-virus interaction (Rivera-Toledo et al., 2015). Therefore, at 24 h post-infection, the virus may have completed a significant portion of its replication cycle, leading to changes in host cell signaling and immune responses. In order to eliminate these confounding factors, we applied equal amount of IFN-α to stimulate macrophages in following experiments. The decreased expression of interferon-stimulated genes (ISGs) in RIOK3-KO macrophages further supports the role of RIOK3 in the downstream interferon response pathway. These genes are typically upregulated during viral infections and are integral to the antiviral defense mechanism (Barrat et al., 2019). RIOK3 appears to play a significant role in orchestrating the immune response to RSV infection in macrophages by balancing the production of proinflammatory factors and maximizing the antiviral effects of type I IFNs.

Furthermore, our results as regarding the role of RIOK3 in the JAK1/STAT1 pathway provide new insights into its function in immune signaling. The reduced phosphorylation of JAK1 and STAT1 in RIOK3-KO BMMs upon IFN-α stimulation, and the rescue of this phenotype with RIOK3 overexpression, strongly suggest that RIOK3 is involved in the JAK1/STAT1 signaling pathway. This pathway is a central mediator of the cellular response to type I IFNs (Ivashkiv and Donlin, 2014), and our results indicate that RIOK3 is required for the proper activation of this pathway following RSV infection. The interaction between RIOK3 and JAK1/STAT1 pathway components, as shown by immunoprecipitation, provides a potential mechanism for RIOK3′s role in modulating this pathway. However, a limitation of our study is the weak pulldown signal of RIOK3 by JAK1 immunoprecipitation, suggesting that the interaction between these proteins, as well as the interaction between kinase-dead mutant RIOK3 and JAK1, should be further explored.

The enhanced viral replication, increased interferon production, but reduced interferon response in the absence of RIOK3 suggest that RIOK3 may be a host factor that RSV has evolved to counteract. This could potentially occur through the modulation of the JAK1/STAT1 pathway, a key regulator of the antiviral process. Our data showed that RIOK3 expression increased at early time points but declined at later stages of RSV infection in THP-1 cells, both at the mRNA and protein levels. The ability of RSV to infect macrophages and potentially manipulate RIOK3 expression could have implications for the virus's capacity to evade the host immune response. Given RIOK3′s role in modulating the interferon response and its impact on RSV replication, RIOK3 could be a potential target for therapeutic intervention. Modulating RIOK3 activity or expression could enhance the host's antiviral response to RSV, providing a new avenue for the development of treatments for RSV infections.

## 5 Conclusion

In conclusion, our study provides evidence that RIOK3 is a critical component of the macrophage response to RSV infection, playing a role in regulating type I IFNs production and the JAK1/STAT1 pathway. The absence of RIOK3 results in enhanced viral replication and a diminished interferon response, highlighting its importance in the host defense against RSV infection. Further research is needed to fully understand the complex interplay between RIOK3, the JAK1/STAT1 pathway, and RSV, which could potentially lead to novel therapeutic strategies against RSV infections.

## Data Availability

The original contributions presented in the study are included in the article/Supplementary material, further inquiries can be directed to the corresponding author.

## Supplementary material

Supplementary Table 1 Detailed information of reagent and resource used in research.

Supplementary Figure 1 *In-vivo* experiments didn't show an increase of RSV replication and Type I IFNs production, as well as inflammatory factors. Control or RIOK3^−/−^ mice were intranasal infected with RSV titer 1 × 10^7^ pfu/mouse. Lung tissue was collected for RNA extraction and detect RSV-F, IFNs and proinflammatory factors by qRT-PCR on day 3 and 5 post infection **(a, b)**. Lavage post 3 day infection was collected for IFNs ELISA assay **(c, d)**. Also, lung tissues were fixed in 10% buffered formalin followed by paraffin embedding for more than 48 h and cross-sections were stained with hematoxylin and eosin (H&E). Light microscopy was applied to record picture on 4X and 40X scale **(e)**.
