# Supplementary figures and images for: The economical role of RIOK3 in modulating the Jak1/STAT1 pathway and antiviral immunity against respiratory syncytial virus infection in macrophages: implications for therapeutic potential
Source: Front Microbiol. 2025 Apr 30;16:1591473. doi: 10.3389/fmicb.2025.1591473 (PMC12075202; doi:10.3389/fmicb.2025.1591473)

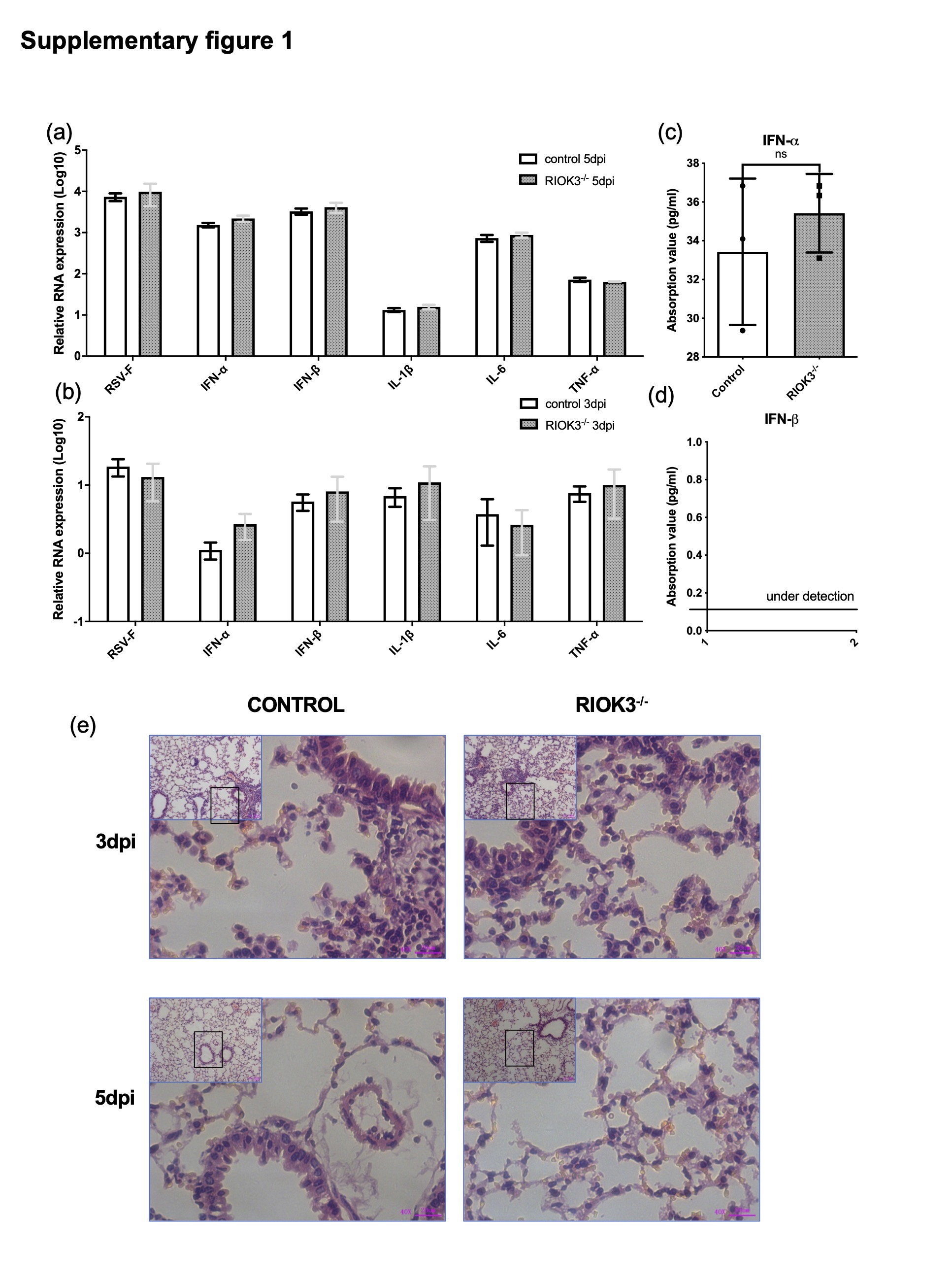

Supplement: Supplementary Figure 1 — In-vivo experiments didn't show an increase of RSV replication and Type I IFNs production, as well as inflammatory factors. Control or RIOK3−/− mice were intranasal infected with RSV titer 1 × 10^7^ pfu/mouse. Lung tissue was collected for RNA extraction and detect RSV-F, IFNs and proinflammatory factors by qRT-PCR on day 3 and 5 post infection (a, b). Lavage post 3 day infection was collected for IFNs ELISA assay (c, d). Also, lung tissues were fixed in 10% buffered formalin followed by paraffin embedding for more than 48 h and cross-sections were stained with hematoxylin and eosin (H&E). Light microscopy was applied to record picture on 4X and 40X scale (e). [file Presentation_1.zip › supplementary Figure 1.jpg]
